# Apathy and Reduced Speed of Processing Underlie Decline in Verbal Fluency following DBS

**DOI:** 10.1155/2017/7348101

**Published:** 2017-03-20

**Authors:** Jennifer A. Foley, Tom Foltynie, Ludvic Zrinzo, Jonathan A. Hyam, Patricia Limousin, Lisa Cipolotti

**Affiliations:** ^1^National Hospital for Neurology and Neurosurgery, Queen Square, London, UK; ^2^UCL Institute of Neurology, Queen Square, London, UK; ^3^Dipartimento di Scienze Psicologiche, Pedagogiche e della Formazione, Università degli Studi di Palermo, Palermo, Italy

## Abstract

*Objective*. Reduced verbal fluency is a strikingly uniform finding following deep brain stimulation (DBS) for Parkinson's disease (PD). The precise cognitive mechanism underlying this reduction remains unclear, but theories have suggested reduced motivation, linguistic skill, and/or executive function. It is of note, however, that previous reports have failed to consider the potential role of any changes in speed of processing. Thus, the aim of this study was to examine verbal fluency changes with a particular focus on the role of cognitive speed. *Method*. In this study, 28 patients with PD completed measures of verbal fluency, motivation, language, executive functioning, and speed of processing, before and after DBS. *Results*. As expected, there was a marked decline in verbal fluency but also in a timed test of executive functions and two measures of speed of processing. Verbal fluency decline was associated with markers of linguistic and executive functioning, but not after speed of processing was statistically controlled for. In contrast, greater decline in verbal fluency was associated with higher levels of apathy at baseline, which was not associated with changes in cognitive speed. *Discussion*. Reduced generativity and processing speed may account for the marked reduction in verbal fluency commonly observed following DBS.

## 1. Introduction

Deep brain stimulation of the subthalamic nucleus (STN DBS) can offer marked improvements in motor function for people with Parkinson's disease (PD), with significant reductions in tremor, rigidity, and bradykinesia (e.g., [1]). DBS has been shown to be very safe, with few serious adverse events occurring either during or following surgery (see review by [2]).

Studies have in general reported very little change in cognitive functioning following DBS ([3–7]), except for one strikingly common and persistent finding: reduced verbal fluency, with significant reductions reported in both letter- and category-based word generation tasks (see reviews by [8] and [9]). The decline occurs very shortly after surgery, with deterioration documented after as little as one month [10] but with minimal further change over time [4, 11]. Although such deterioration can occur as part of the natural progression of PD, the loss experienced is greatly exacerbated following DBS (see review by [12]).

The acute onset of this decline has led some researchers to consider whether the surgery or the subsequent stimulation itself may be causing this difficulty, perhaps by disrupting neural circuits underlying verbal fluency [13]. Indeed, one PET study found reduced activation of the left frontotemporal network during a verbal fluency task in seven patients with advanced PD and STN stimulation [14]. In contrast, other studies with larger sample sizes have failed to elicit any association with stimulation parameters (e.g., [15, 16]) and a meta-analysis of 40 studies revealed no relationship between changes in verbal fluency and DBS stimulation parameters [9].

Thus, the cause of this deterioration remains unclear. Several theories have been proposed, but it may be argued that previous studies have failed to consider basic speed of processing as a causal or confounding factor. Although processing speed is known to decline with the onset of PD [17], most studies investigating post-DBS cognitive changes have tended to omit any measure of processing speed [9], which hitherto has obscured its role in the decline in verbal fluency. Therefore, each of the putative theories will now be discussed, with special consideration given to the possible role of speed of processing.

### 1.1. Increase in Apathy

Some studies have found a correlation between decline in verbal fluency and emergence of apathy following surgery [4, 15]. Apathy is the reduction of motivation [18], which can manifest as decreased interest, emotional responsivity, and/or goal-related behaviour. Although common in PD [19], apathy increases following DBS [20–24], which may reflect the withdrawal of dopaminergic medication following DBS [25–27] or the procedure itself [22].

Only a handful of studies have examined the relationship between decline in verbal fluency following DBS and emergence of apathy, with some reporting significant associations [4, 15, 28].

Apathy in PD has been found to be associated with greater cognitive inefficiency in general and in particular slower speed of processing [29]. Of the few studies that have explored the relationship between verbal fluency and apathy after DBS, only one also assessed changes in speed of processing. Interestingly, this revealed significant declines not only in verbal fluency but also in speed of processing [30]. As speed was not considered as a potential covariate, it remains unclear how much of the relationship between postoperative apathy and decline in verbal fluency can be explained by declines in basic speed of processing.

### 1.2. Reduced Word Knowledge

Verbal fluency is also dependent upon word knowledge, with deterioration in naming associated with reductions in fluency (e.g., [31]). Some studies have also suggested that linguistic functioning may be compromised in PD, particularly affecting action-verb processing [32–35], and it has been proposed that the frontostriatal circuits affected by PD may also have a role in the processing of action verbs within the motor cortex [36].

Whelan et al. [37, 38] examined a range of language functions before and after STN DBS and found evidence of reduced semantic processing. Such changes may explain the observed reduction in verbal fluency, reflecting the reduced pool of exemplars to select from. However, closer examination reveals that the purported reductions in semantic processing refer to slower reaction times on a lexical decision task. This was not limited to specific types of words and was simply a slower version of the same pattern observed before DBS. Therefore, the pattern of findings could also be explained by a general pattern of postoperative slowing. A recent study by Ehlen et al. [39] also suggested that any postoperative linguistic deficits, including reduced verbal fluency, may simply reflect reduced speed of processing.

### 1.3. Greater Executive Dysfunction

Another hypothesis is that the reduction in verbal fluency reflects deterioration in executive functions. Executive functions are higher-order cognitive processes, which include focusing and maintenance of attention, inhibition of inappropriate behaviours, and set-switching (e.g., [40–44]). Executive functions have been argued to support verbal fluency in the effortful self-initiation of performance, efficient organisation of word retrieval and recall, switching between different retrieval strategies, and self-monitoring of exemplars generated [45, 46]. These executive processes may be most active after the initial 15–20 seconds of a verbal generation task, during which production of words is fairly automatic, relying upon easily accessible and commonly used words. After this initial phase, subsequent verbal generation is thought to involve more effortful and controlled processing and thus greater executive control [47].

Declines in verbal fluency are often accompanied by deterioration in executive functions (e.g., [4, 30, 48]). Troyer [49] argued that optimal fluency performance involves the generation of words within a subcategory and that when a subcategory is exhausted, switching to a new subcategory. Executive dysfunction is thought to lead to fewer successful switches, with reduced retrieval of appropriate subcategories and fewer exemplars generated in total [50–52]. In contrast, generation of exemplars, or “clusters”, within a category, is thought to rely upon semantic memory stores [50, 53, 54] and be relatively unaffected by executive dysfunction.

In keeping with this, there are reports of reductions in switching, but not clustering following STN DBS [15, 55]. This would suggest that a deficit in cognitive set-shifting underlies the decline in verbal fluency. Yet, several studies have reported verbal fluency reductions in the absence of any deficits on other measures of set-shifting (e.g., [4, 20, 56]) and in fact occasionally in the presence of significant improvements on other measures of set-shifting, such as the Trail-Making Test, part B (TMT-B; [57–59]), and the Wisconsin Card Sorting Test [11].

In contrast, these declines in verbal fluency are often accompanied by reduced motor and/or processing speed [30]. It is possible that fewer switches generated simply reflect the longer time it takes to generate responses: leading to a reduction in the number of switches (within a specific time frame), but no changes in the number of exemplars generated within any given cluster. For example, Saint-Cyr et al. [55] reported that the observed reduction in verbal fluency was accompanied by an increase in time taken to perform the TMT-B, which they suggest reflects a common decline in executive functioning. However, they also report a sizeable increase in mean time taken to perform the TMT-A, suggestive of a more basic slowing of speed of processing.

Thus, although there is evidence that apathy, word knowledge, and executive functions may be involved in the decline in verbal fluency observed after DBS, the role of speed of processing remains to be explored. The aim of this study was to examine the relationship between verbal fluency and apathy and word knowledge and executive functions and to consider the role of speed of processing in a group of PD patients undergoing DBS.

## 2. Methods

### 2.1. Participants

A total of 28 patients (17 male, 11 female) took part in this study. All patients had a diagnosis of idiopathic PD, according to Queen Square Brain Bank criteria, for at least five years, were younger than 70 years old, and suffered from disabling motor complications despite optimal treatment. Each patient underwent multidisciplinary evaluation to decide on suitability. A formal levodopa challenge confirmed dopaminergic drug responsiveness. Detailed neuropsychological and neuropsychiatric assessments excluded patients with significant cognitive impairment and/or ongoing psychiatric comorbidities. A structural MRI was obtained to exclude surgical contraindications. Patient characteristics are found in Table 1.

All patients were assessed pre- and postsurgery under their optimal conditions. Thus, preoperatively, this was on medication and postoperatively on medication and stimulation. The second assessment was performed with a mean of 19.50 months after surgery (range = 1–54; SD = 12.13).

### 2.2. Neuropsychological Assessment

All of the participants completed a battery of standardised cognitive and mood assessments. The tests chosen assessed the following.

#### 2.2.1. Verbal Fluency

Verbal fluency was assessed using the letter and category subtests from the Delis-Kaplan Executive Functioning System (DKEFS; [60]).

#### 2.2.2. Cognitive Functioning: General Cognition, Memory, and Visual Processing

The MMSE was administered as a screen of global cognitive functioning [61]. Current level of intellectual functioning was assessed using tasks from the Wechsler Adult Intelligence Scale—Third Edition (WAIS-III; [62]), prorated to generate scores for both verbal (VIQ) and nonverbal intellectual abilities (PIQ). The National Adult Reading Test (NART; [63]) was used in order to estimate the premorbid level of intellectual functioning, by generating each patient's Predicted Full-Scale IQ (PFSIQ). Visual and verbal recognition memory were assessed using the Words and Faces Recognition Memory Tests (RMT; [64]). Visual and verbal recall memory were assessed using the Shapes and People subtests from the Doors and People Test (D&P; [65]). Visual processing was assessed using the Silhouettes subtest from the Visual Object and Space Perception Battery (VOSP; [66]).

#### 2.2.3. Apathy

Apathy was assessed using the Apathy Evaluation Scale (AES; [67]), as validated for use in PD by Pluck & Brown [19]. Patients were also screened for mood disorder using the Hospital Anxiety and Depression Scale (HADS; [68]).

#### 2.2.4. Word Knowledge

Word knowledge was assessed using the Graded Naming Test (GNT; [69]) and the Vocabulary subtest from the WAIS-III [62].

#### 2.2.5. Executive Functioning

Executive functioning was assessed using the Stroop [70], Hayling Sentence Completion Test [71], Brixton Spatial Anticipation Test [71], Elevator Counting and Distraction subtests from the Test of Everyday Attention (EC, EC-D; [72]), Modified Card Sorting Test (MCST; [73]), and Trail Making Test (TMT-B/A; [74]).

#### 2.2.6. Speed of Processing

Speed of information processing was assessed using the Symbol Search (SS) and Digit Symbol Coding (DSC) subtests from the WAIS-III [62].

The research was done in accordance with the Helsinki declaration and the Institute of Neurology Joint Research Ethics Committee UCLH, NHS Trust Research and Development Directorate.

### 2.3. Statistical Analysis

Where possible, raw scores were transformed into scaled scores, with reference to the available normative data described in the manuals for each of the measures listed. Mean and standard deviations were calculated for each of the variables pre- and post-DBS. Normality of distribution was assessed using the Kolmogorov-Smirnov test and, if significant, by examining the *z*-scores for skewness and kurtosis. Homogeneity of variance was assessed using Levene's test. Unless otherwise stated, all data met the assumptions of normality and homogeneity of variance. Pre- and post-DBS scores were compared using *t*-tests for related samples or Wilcoxon signed-ranks, as appropriate. The relationship between decline in verbal fluency and other scores was investigated using Pearson and Spearman correlational analyses, with partial correlations used when seeking to control the effect of any potential covariates. Finally, a backwards regression analysis was conducted to determine the predictive value of any correlates of decline in fluency. Post hoc analyses were adjusted for multiple comparisons using Bonferroni correction. All analyses were conducted using IBM SPSS Statistics Data Editor version 19.

## 3. Results

### 3.1. Motor Performance Pre- and Post-DBS

As shown in Table 2, there was a significant improvement in motor status, as assessed by part III of the Unified Parkinson's Disease Rating Scale (UPDRS-III), off medication following DBS (*t* (23) = 6.50, *p* < .001), but there were no significant changes on medication. Significant changes are highlighted in bold.

### 3.2. Cognitive Performance Pre- and Post-DBS

Table 3 reveals cognitive test scores at baseline and following surgery, with all significant changes highlighted in bold.

### 3.3. Decline in Cognition following DBS

As shown in Table 3, paired sample *t*-tests revealed decline in verbal fluency after DBS. Performance deteriorated significantly on both measures of letter (*t* (25) = 2.74, *p* < .05) and category fluency (*t* (25) = 3.67, *p* < .05).

In addition to the decline noted in verbal fluency, the patients also demonstrated a significant decline on two further timed measures of cognitive functioning, namely TMT-B/A (*Z* = −2.26, *p* < .05) and SS (*t* (25) = 2.44, *p* < .05).

### 3.4. Potential Cognitive Mechanisms Underlying Verbal Fluency Decline

#### 3.4.1. Increase in Apathy

*(1) Pre- and Post-DBS Ratings.* At baseline, around a third of patients endorsed clinical levels of apathy (*n* = 9, 32.1%). In addition, many patients also had scores indicative of depression (*n* = 8, 28.6%) or anxiety (*n* = 15, 53.6%).

As shown in Table 4, paired *t*-tests and Wilcoxon signed-ranks revealed no significant changes in mean ratings of apathy, anxiety, or mood after DBS.

*(2) Relationship between Verbal Fluency Decline and Apathy.* Pearson correlational analyses revealed that higher levels of apathy at baseline were associated with greater decline in category fluency after DBS (*r* = −.44, *p* < .05).

As shown in Table 5, there were no correlations between change in apathy and change in verbal fluency following DBS. Significant relationships are highlighted in bold.

*(3) Role of Speed of Processing.* As shown in Table 5, Pearson correlational analyses revealed significant correlations between decline in category fluency and decline in processing speed, as measured by both SS (*r* = −.53, *p* < .05) and DSC (*r* = −.45, *p* < .05) following DBS. Similarly, greater increases in apathy were correlated with greater decline in SS following DBS (*r* = −.45, *p* < .05).

A partial correlation revealed that even when performance on SS and DSC was statistically controlled for, there remained a significant correlation between greater decline in category fluency and higher levels of apathy at baseline (*r* = −.51, *p* < .05) after DBS.

#### 3.4.2. Reduced Word Knowledge

*(1) Pre- and Post-DBS Performance.* As shown in Table 3, there were no significant changes in performance on either the GNT or Vocabulary subtest after DBS.

*(2) Relationship between Verbal Fluency Decline and Word Knowledge.* Pearson and Spearman correlational analyses revealed no significant relationship between baseline performance on the NART, GNT, or Vocabulary subtest and subsequent decline in verbal fluency after DBS.

However, as shown in Table 5, greater decline in category fluency was associated with greater decline in Vocabulary following DBS. Significant relationships are highlighted in bold.

*(3) Role of Speed of Processing.* There was no significant relationship between change in GNT performance and change in either of the measures of speed of processing. However, decline in Vocabulary was significantly associated with SS decline (*r* = .41, *p* < .05). When performance on SS was statistically controlled for, there was no longer a significant correlation between the decline in category fluency and Vocabulary after DBS.

#### 3.4.3. Greater Executive Dysfunction

*(1) Pre- and Post-DBS Performance.* As shown in Table 3, there was a significant reduction in performance on one measure of executive functioning following DBS, namely TMT-B/A.

Table 6 shows the verbal fluency parameters of words generated during letter and category fluency tasks before and after DBS, with significant changes highlighted in bold.

As shown in Table 6, there was a significant drop in the number of words produced in the second time interval (16–60 seconds) of the letter fluency task following STN DBS.

Table 7 shows the correlations between decline in verbal fluency and verbal fluency parameters before and after DBS, with all significant relationships highlighted in bold.

As seen in Table 7, the drop in verbal fluency observed was not selective to the size of cluster or the total number of switches made. Moreover, the decline in letter fluency was significantly correlated with declines in both time intervals: 0–15 seconds and 16–60 seconds.

*(2) Relationship between Verbal Fluency Decline and Executive Functioning.* A series of Pearson and Spearman correlational analyses revealed no significant associations between baseline executive scores and decline in either letter or category fluency after DBS.

Correlational analyses examining the relationship between baseline executive scores and decline in the verbal fluency parameters revealed a number of significant correlations. Specifically, poorer baseline performance on the Stroop was associated with greater decline in mean cluster size (*r* = .51, *p* < .05) and increase in the mean number of switches (*r* = −.55, *p* < .05) during letter fluency tasks following DBS. Similarly, poorer baseline performance on the Brixton was associated with an increase in the mean number of switches during the category fluency task after DBS (*r* = −.46, *p* < .05). Furthermore, poorer baseline performance on ECD was associated with greater decline in performance during the 16–60-second time interval (but not during 0–15 seconds) of the letter fluency tasks (*r* = −.56, *p* < .05) following DBS. However, none of these correlations remained statistically significant after Bonferroni correction for multiple comparisons.

Decline in letter fluency was significantly correlated with decline in performance on the Hayling after DBS (*p* = −.50, *p* < .05). Decline in category fluency was also associated with decline in performance on the Hayling (*p* = −.47, *p* < .05) and the Stroop (*p* = −.40, *p* < .05) after DBS.

Correlational analyses also revealed a number of significant associations between change in executive scores and change in verbal fluency parameters. Greater increase in TMT-B/A score was associated with greater decline in mean cluster size during the category fluency task (*r* = .56, *p* < .05) following DBS. Similarly, greater decline on the Brixton was associated with greater increase in the mean number of switches during the category fluency task (*r* = −.46, *p* < .05) after DBS. Greater decline on both the Stroop and the ECD tasks was associated with greater deterioration in letter fluency during the 16–60-second time interval (Stroop: *r* = .57, *p* < .05; ECD: *r* = .67, *p* < .05), after DBS. However, none of these remained statistically significant after Bonferroni correction for multiple comparisons.

*(3) Role of Speed of Processing.* Pearson correlations revealed no significant correlations between decline in executive functioning and decline in speed of processing after either DBS. Greater decline in category fluency was associated with greater decline on both SS and DSC, but no correlation was found between speed of processing and decline in either the cluster size or the number of switches after DBS. However, greater slowing on SS was significantly associated with greater decline on both letter (*r* = .53, *p* < .05) and category fluency during the 16–60-second time interval (*r* = .65, *p* < .01), with the latter remaining significant after adjustment for multiple comparisons.

Strikingly, partial correlations revealed that when performance on SS was statistically controlled for, no significant correlations remained between change on any of the measures of executive functioning and change on any measure of verbal fluency DBS.

### 3.5. Predictors of Verbal Fluency Decline

In sum, decline in category fluency following DBS was correlated with greater apathy at baseline, greater change in performance on the Vocabulary, Stroop and Hayling tests, and greater slowing on the SS and DSC subtests. In order to determine the predictive value of each of these, a multiple regression analysis was conducted. Using a backwards method, a significant model emerged (*F*_2,22_ = 9.89, *p* < .01), revealing that change on the Stroop and DSC together accounted for 43.9% of the variance. Baseline level of apathy and change in Vocabulary, Hayling, or SS were not significant predictors.

## 4. Discussion

Several studies have reported significant declines in letter- and category-based verbal fluency following DBS (see [9]), and this was also found in the current study. The major goal of this study was to identify the mechanism underlying this decline. Previous reports have suggested that it may be explained by an increase in apathy [4, 15], decline in word knowledge [37, 38], and/or greater executive dysfunction [50–52], but crucially, these earlier studies have failed to consider how the basic speed of processing may also contribute.

### 4.1. Apathy

In this study, DBS patients presented with a similarly high rate of apathy at baseline (32.1%). This higher level of apathy at baseline was significantly correlated with greater subsequent decline in category fluency, supporting earlier studies describing a link between apathy and reduced verbal fluency. Interestingly, although verbal fluency deteriorated following DBS, we found no significant change in the level of apathy reported. This deviates from previous findings; for example, Martinez-Fernandez et al. [75] found that none of their 102 patients demonstrated a clinical level of apathy at baseline, but 27.1% did so 12 months after DBS. It is possible that these conflicting findings may be explained by the present patients demonstrating very high rates at baseline, thus creating a type of ceiling effect preventing any further significant change.

Alternatively, these differences may be explained by variation in assessment methods; the current study used a self-rated rather than clinician-rated measure, unlike many of the previous studies (Antonini et al., 2011; [4, 15]). Our findings are in line with those of Castelli et al. [20], whom also found no relationship between changes in verbal fluency and apathy and who also used a self-rated measure of apathy. Indeed, Schiehser et al. [76] emphasise the importance of considering the identity of the reporter when using subjective measures of apathy.

The lack of significant change may also be because of differences in medication changes postoperatively. It is possible that in this study, there was a more judicious reduction in dopaminergic medication than in previous studies [25, 27], limiting the emergence of any postoperative apathy. Unfortunately, this study did not have sufficient data on medication dosages pre- and post-DBS to investigate this, but future studies may wish to explore this.

When considering the role of speed of processing, it is noted that postoperative increase in apathy was associated with a greater decline in speed of processing. Irrespectively, there remained a relationship between baseline levels of apathy and subsequent decline in category fluency even after speed was factored out. This hints at a shared cognitive mechanism underlying the decline in generativity seen in both apathetic syndromes and diminished verbal fluency, which cannot be explained by slowed speed of processing.

It is possible that this relationship reflects the onset of a more general cognitive decline. Apathy has been identified as a harbinger of PD dementia [29, 77] and several reports have revealed higher rates of apathy in PD patients with dementia [78, 79]. The link between apathy and cognitive impairment has been explained by a diffuse pathophysiology. Recently, apathy and dementia have been associated with greater amyloidopathy and amyloid load in PD [80, 81], and anticholinergics have been found to be useful in the treatment of both cognitive impairment and apathy in advanced PD [82]. Thus, there is mounting evidence to support the link between apathy and incipient dementia, and future studies should examine if patients with greater apathy at baseline who experience reduced verbal fluency after DBS are in turn more vulnerable to PD dementia.

### 4.2. Word Knowledge

Word knowledge at baseline was not associated with subsequent reductions in verbal fluency, but decline in Vocabulary was associated with greater decline in category fluency after DBS. At first glance, this finding appears to support earlier studies that have suggested that linguistic abilities are compromised in PD, particularly affecting semantic processing [37, 38]. Yet, change in Vocabulary was also found to be related to speed of processing, and when we statistically controlled for this, there was no longer a significant correlation between category fluency and Vocabulary performance. This is in line with earlier studies [37, 38] that have shown that the purported reduction in semantic processing in fact referred to slowed reaction times on a lexical decision task. These findings would suggest that any relationship between verbal fluency and linguistic processing observed may simply reflect a general reduction of cognitive speed.

### 4.3. Executive Functioning

Baseline performance on measures of executive functioning was not associated with changes in verbal fluency. There was a significant reduction in performance on TMT-B/A after DBS, and a decline on both Stroop and Hayling was associated with reduced fluency after DBS. However, after controlling for changes in speed of processing, there were no longer any significant correlations between change in category fluency and any measure of executive functioning. The predictive value of the Stroop may instead refer to its emphasis upon speeded processing. The other only significant predictor of verbal fluency decline was DSC, which suggests that the change in fluency relies greatly upon change in speed of processing.

Finer-grained analysis revealed that lower baseline executive scores were associated with greater decline in the number of clusters and increased the number of switches, as well as the performance during the 16–60-second time interval of fluency tasks. In addition, greater reduction on measures of executive functioning was associated with increase in the number of switches and performance during the 16–60-second time interval. This confirms the executive load of both switching [50–52] and generation of exemplars after the initial cueing [47]. However, reduction in verbal fluency was selective to neither the number of switches nor the time frame. Therefore, it would appear that the decline in verbal fluency does not simply reflect deterioration in executive functioning, but rather a more general reduction in cognitive speed.

### 4.4. Overall Conclusion

Although dysfunction of frontostriatal circuitry is known to lead to gradual decrements in speed of processing in PD [17], our study has shown that processing speed is significantly reduced following DBS and is the most important predictor of decline on tests of verbal fluency. This finding suggests a degree of frontostriatal dysfunction that is not caused by disease progression alone [83, 84].

Frontostriatal circuitry connects the STN to frontal associative areas [83], including the dorsomedial prefrontal cortex [85]. The dorsomedial prefrontal cortex has been associated with speed of processing [86], and specifically “energization” [44]. Stuss and colleagues have argued that energization is required for establishing a response mode and initiating output, with deficits leading to slowed response [86], reduced verbal fluency [87], and apathy [43]. Thus, in theoretical terms, this pattern of deficits is consistent with a deficient “energization” function of the frontal attentional system [86–88]. Future studies may wish to consider further the impact of DBS on energization and its role in PD cognitive decline.

## Figures and Tables

**Table 1:** Patient characteristics.

	Mean ± SD
Age (at first assessment; years)	57.50 ± 7.32
NART-predicted IQ	111.57 ± 11.08
Age at PD diagnosis (years)	45.55 ± 7.80
PD disease duration (years)	18.77 ± 6.12

**Table 2:** Motor symptoms pre- and post-DBS.

	Pre-DBS	Post-DBS	*p*
UPDRS-III off med	**48.68** ± **14.10**	**28.67** ± **9.99**	<.001^∗^
UPDRS-III on med	17.29 ± 7.97	15.83 ± 7.20	.49

Results are given as mean ± SD (^∗^*p* < .05). UPDRS-III: Unified Parkinson's Disease Rating Scale, part III.

**Table 3:** Cognitive performance pre- and post-DBS.

	Pre-DBS	Post-DBS	*p*
Letter fluency (SS)	**13.42** ± **4.89**	**11.54** ± **4.61**	**.01** ^a^^∗^
Category fluency (SS)	**12.31** ± **4.21**	**10.00** ± **4.99**	**.01** ^a^^∗^
MMSE (/30)	28.64 ± 1.41	28.64 ± 1.68	1.00^a^
WAIS-VIQ	111.21 ± 12.40	107.54 ± 15.93	.05^a^
WAIS-PIQ	106.37 ± 15.17	104.04 ± 19.57	.50^a^
RMT-words (/50)	46.81 ± 3.50	45.12 ± 5.35	.10^b^
RMT-faces (/50)	41.88 ± 4.13	41.08 ± 5.68	.54^a^
D&P-people delayed (/12)	7.21 ± 3.68	7.50 ± 4.30	.59^b^
D&P-shapes delayed (/12)	10.50 ± 3.28	10.25 ± 2.82	.22^b^
VOSP-silhouettes (/30)	22.81 ± 3.25	21.92 ± 3.91	.13^a^
GNT (/30)	23.69 ± 3.42	23.69 ± 3.28	1.00^a^
Stroop (/112)	91.81 ± 21.36	83.77 ± 22.94	.06^a^
Vocabulary (SS)	12.71 ± 2.73	12.29 ± 2.61	.18^a^
Hayling (SS)	5.68 ± 1.07	5.32 ± 1.52	.28^b^
Brixton (SS)	4.91 ± 1.53	5.00 ± 2.28	.83^a^
TEA EC (/7)	6.67 ± 0.96	6.75 ± 0.44	.85^b^
TEA ECD (SS)	9.91 ± 2.66	8.96 ± 2.92	.15^a^
MCST-categories (/6)	5.39 ± 1.16	5.17 ± 1.56	.55^b^
TMT-B/A	**2.32** ± **0.78**	**2.79** ± **1.02**	**.02** ^b^^∗^
SS	**9.62** ± **2.25**	**8.46** ± **2.82**	**.02** ^a^^∗^
DSC (SS)	8.20 ± 2.52	7.48 ± 2.65	.22^a^

Results are given as mean ± SD (^a^paired *t*-test, ^b^Wilcoxon signed-rank, ^∗^*p* < .05). WAIS-III: Wechsler Adult Intelligence Scale—Third Edition [62]; RMT: Recognition Memory Test [64]; D&P: Doors and People (Baddeley, Emslie & Nimmo-Smith, 2006); VOSP: Visual Object and Space Perception Battery [66]; GNT: Graded Naming Test [69]; Stroop [70]; Vocabulary, WAIS-III [62]; Hayling: Hayling Sentence Completion Test [71]; Brixton: Brixton Spatial Anticipation Test [71]; TEA EC: Test of Everyday Attention, Elevator Counting [72]; TEA ECD: Test of Everyday Attention, Elevator Counting with Distraction [72]; MCST: Modified Card Sorting Test [73]; TMT: Trail Making Test [74]; SS: Symbol Search, WAIS-III [62]; DSC: Digit Symbol Coding, WAIS-III [62].

**Table 4:** Apathy and mood ratings pre- and post-DBS.

	Pre-DBS	Post-DBS	*p*
AES-apathy	10.75 ± 6.02	13.96 ± 11.16	.15^b^
HADS-depression	6.15 ± 4.42	6.00 ± 4.04	.86^a^
HADS-anxiety	7.50 ± 3.23	6.27 ± 4.85	.19^b^

Results are given as mean ± SD (^a^paired *t*-test, ^b^Wilcoxon signed-rank). AES: Apathy Evaluation Scale [67]; HADS: Hospital and Anxiety Depression Scale [68].

**Table 5:** Correlations between decline in verbal fluency and changes in other measures after DBS.

	Letter fluency	Category fluency
AES	.15^a^	.33^a^
GNT	−.04^a^	−.04^a^
Vocabulary	−.32	−**.45**^a^^∗^
Stroop	−.26^a^	−**.40**^a^^∗^
Hayling	−**.50**^b^^∗^	−**.47**^b∗^
Brixton	−.09^a^	−.20^a^
TEA ECD	−.23^a^	−.27^a^
MCST-categories	−.16^b^	−.08^b^
TMT-B/A	.07^a^	−.04^a^
SS	−.32^a^	−**.53**^a^^∗^
DSC	.14^a^	−**.45**^a^^∗^

^a^Pearson's *r* coefficient, ^b^Spearman's *p* coefficient, ^∗^*p* < .05. AES: Apathy Evaluation Scale [67]; GNT: Graded Naming Test [69]; Vocabulary, WAIS-III [62]; Stroop [70]; Hayling: Hayling Sentence Completion Test [71]; Brixton: Brixton Spatial Anticipation Test [71]; TEA ECD: Test of Everyday Attention, Elevator Counting with Distraction [72]; MCST: Modified Card Sorting Test [73]; TMT: Trail Making Test [74]; SS: Symbol Search, WAIS-III [62]; DSC: Digit Symbol Coding, WAIS-III [62].

**Table 6:** Letter and category fluency characteristics pre- and post-DBS.

	Letter fluency (F, A, S)			Category fluency (animals)		
	Pre-DBS	Post-DBS	*p*	Pre-DBS	Post-DBS	*p*
Words: 0–15 s	18.13 ± 6.14	16.07 ± 6.53	.06^a^	15.67 ± 3.75	14.13 ± 5.04	.15^a^
Words: 16–60 s	27.73 ± 11.46	28.07 ± 15.17	.87^a^	27.20 ± 9.13	23.13 ± 9.09	**.04** ^a^^∗^
Cluster size	3.31 ± 2.14	2.98 ± 1.55	.50^a^	1.27 ± 0.88	1.59 ± 1.01	.29^b^
Number of switches	23.68 ± 14.29	21.18 ± 12.37	.32^a^	8.86 ± 6.92	7.27 ± 4.71	.28^a^
Total errors	2.68 ± 2.23	1.64 ± 1.59	.05^a^	0.77 ± 1.06	0.55 ± 0.96	.50^b^

Results are given as mean ± SD (^a^paired *t*-test, ^b^Wilcoxon signed-rank, ^∗^*p* < .05).

**Table 7:** Pearson correlations between decline in verbal fluency and fluency characteristics after DBS.

	Letter fluency	Category fluency
Words: 0–15 s	.59^∗^	.58^∗^
Words: 16–60 s	.75^∗^	.74^∗^
Cluster size	−.15	.32
Number of switches	.27	.25
Total errors	.12	−.17

^∗^ *p* < .05.
